# We are not alone: a case for the human microbiome in extra intestinal diseases

**DOI:** 10.1186/s13099-017-0163-3

**Published:** 2017-03-07

**Authors:** S. Shivaji

**Affiliations:** Prof. Brien Holden Eye Research Centre, Prof D Balasubramanian Chair of Research, L V Prasad Eye Institute, Road No 2, Banjara Hills, Hyderabad, Telangana 500 034 India

**Keywords:** Microbiome, Dysbiosis, Disease

## Abstract

**Background:**

“Dysbiosis” in the gut microbiome has been implicated in auto-immune diseases, in inflammatory diseases, in some cancers and mental disorders. The challenge is to unravel the cellular and molecular basis of dysbiosis so as to understand the disease manifestation.

**Main body:**

Next generation sequencing and genome enabled technologies have led to the establishment of the composition of gut microbiomes and established that “dysbiosis” is the cause of several diseases. In a few cases the cellular and molecular changes accompanying dysbiosis have been investigated and correlated with the disease. Gut microbiome studies have indicated that *Christensenella minuta* controls obesity in mice, *Faecalibacterium prausnitzii* protects mice against intestinal inflammation and *Akkermansia muciniphila* reverses obesity and insulin resistance by secreting endocannabinoids. In mice polysaccharide antigen A on the surface of *Bacteroides fragilis*, reduces inflammation. Such experiments provide the link between the gut microbiome and human health but implicating dysbiosis with extra-intestinal diseases like arthritis, muscular dystrophy, vaginosis, fibromyalgia, some cancers and mental disorders appears to be more challenging. The relevance of gut microbiome to the eye appears to be very remote. But considering that the eye is the site of inflammatory diseases like uveitis, scleritis, Mooren’s corneal ulcer etc. it is possible that these diseases are also influenced by dysbiosis. In mice signals from the gut microbiota activate retina specific T cells that are involved in autoimmune uveitis. Such information would open up new strategies for therapy where the emphasis would be on restoring the diversity in the gut by antibiotic or specific drug use, specific microbe introduction, probiotic use and fecal transplant therapy. The ocular surface microbiome may also be responsible for eye diseases in man but such studies are lacking. Microbiome of the healthy cornea and conjunctiva have been identified. But whether the ocular microbiome exhibits dysbiosis with disease? Whether ocular microbiome is influenced by the gut microbiome? What mediates the cross-talk between the gut and ocular microbiomes? These are questions that need to be addressed to understand idiopathic infections of the eye.

**Conclusions:**

Evaluating diseases remote from the gut would unfold the mysteries of the microbiome.

## Background

The only time we are alone is prior to birth. Then onwards we are colonized by innumerable number of bacteria acquired from the mother and the environment and by adulthood we have trillions of bacteria colonizing every surface of the body. In the adult gut the number increases to 10^14^ bacteria which weighs around 1.13 kg and is ten times more than the total number of cells in the human body (10^13^). Thus we are more bacteria than human. This enormous community of microorganisms was termed the “microbiome” by Lederberg and Mccray [1] and was defined as a multi-species community of microorganisms (bacteria, fungi and viruses) that differs from niche to niche in population density and community composition. For instance the microbiome of the gut is different from that on the skin, on the scalp, inside the nostrils, mouth, armpits, oesophagus, stomach, small intestine, vagina etc. [2–4]. What could be the reasons for the variation? How is it important? And what is the role of genes associated with the microbiome which according to conservative estimates is 200–300 times more than the total genes in the human body? These are few of the questions that need to be answered.

## Main text

### Dysbiosis and diseases

Till the 1990s, it has been a mystery as to whether the microbes/genes in the gut microbiome have any impact on human health other than the canonical role of the gut bacteria to break down food remnants, modulate the immune system, promote fat storage, biosynthesis of vitamins and amino acids, metabolism of drugs etc. But, with the advent of next generation sequencing and other genome enabled technologies including DNA based databases and metabolomics on human microbiome studies have become an eye-opener to hitherto unknown facts about the composition of gut microbiomes, their dynamics and their relevance to human welfare [5]. At the phylum level *Bacteroidetes* and *Firmicutes* are the most dominant bacteria in the gut of normal individuals and together constitute 70–90% of the total bacterial community followed by *Actinobacteria* and *Proteobacteria*. This community is dynamic and known to vary in normal individuals with age, ethnicity, diet, exposure to chemicals and also host genetic variation [6–9]. Studies analyzing the gut microbiomes of twins established a link between human genotype and the composition of the gut microbiome [7]. All these factors make the very definition of a normal core microbiome a great challenge. Aberrations from the normal core microbiome is referred to as “dysbiosis” (meaning imbalance in the microbiome) and has been implicated in auto-immune diseases like diabetes, rheumatoid arthritis, muscular dystrophy, multiple sclerosis and fibromyalgia, in inflammatory diseases like obesity, neonatal necrotizing enterocolitis, inflammatory bowel disease and vaginosis, in some cancers and mental disorders [10, 11]. Ability of the gut microbiome to modulate the immune system is considered as an important reason for the diseased state [12]. The direct evidence that the microbiota are indeed the cause of the disease is strengthened by the pioneering studies on obesity in mice. Gut bacteria from fat mice when transplanted into genetically lean mice with no gut bacteria of their own, transform the lean mice into obese mice [13, 14]. Subsequent studies demonstrated that skinny germ free mice plump up on receiving a fecal transplant from a human donor implying that the bacteria help the recipient to digest and metabolise more efficiently [13–15]. But, if the fecal transplant of the human donor was supplemented with *Christensenella minuta* the recipient mice were thinner indicating that *C. minuta* controls obesity [7]. Another example, emphasizing the role of gut bacteria is related to *Clostridium difficile* infection (CDI) patients who are cured of CDI when transplanted with fecal microbiota from normal individuals [16, 17]. This led to the concept that just as a pathogen could cause a disease a “good” microbe could prevent a disease? For instance *Faecalibacterium prausnitzii c*ould protect mice against experimentally induced intestinal inflammation since these gut bacteria were anti-inflammatory. Yet another example of a good microbe is *Akkermansia muciniphila.* Increase in abundance of *A. muciniphila* correlated with an improved metabolic profile and reversed obesity and decreased insulin resistance probably mediated by endocannabinoids secreted by *A. muciniphila* [18, 19].

### Dysbiosis and extra-intestinal diseases

Linking food/gut related disease like obesity, inflammatory bowel disease, enterocolitis, diabetes etc. to dysbiosis appears to be obvious but implicating dysbiosis with extra-intestinal diseases like arthritis, muscular dystrophy, multiple sclerosis, vaginosis, fibromyalgia, some cancers and mental disorders appears to be more challenging. These studies indicate that the microbiome has an overarching influence on human health. For instance it was demonstrated that transplanting a specific combination of fecal bacteria into the gut of normal mice rendered them depressed due to changes in the “myelin” sheath that surrounds nerve fibers [20]. Cresol, produced by certain gut bacteria and which reduces the amount of myelin produced by brain cells was higher in mice that became depressed after fecal transplants. It was also observed that in mice modeled on two inflammatory human diseases: colitis and multiple sclerosis, polysaccharide antigen A (PSA) on the surface of the gut microbe *Bacteroides fragilis*, regulates immunity and reduces inflammation [21]. Experiments which produce such leads at the molecular level would help to understand the link between the gut and human health irrespective of the site of pathology and may thus provide leads for therapy.

### Dysbiosis and ocular diseases

The relevance of gut microbiome to the eye appears to be very remote. But considering that the eye is the site of inflammatory diseases like uveitis, scleritis, Mooren’s corneal ulcer etc. which also occur due to autoimmune reaction, it is possible that under non-infectious conditions these diseases are influenced by dysbiosis in the gut. Last year it was demonstrated that in mice with experimentally induced autoimmune uveitis oral administration of antibiotics reduced the severity of uveitis [22]. But, intraperitoneal administration of the same antibiotic did not have the desired effect thus highlighting the role of gut microbiota in affecting uveitis. Simultaneously, it was also observed that the gut microbiome was significantly altered following antibiotic administration. Thus implying that uveitogenic bacteria may exist, and some antibiotics may be able to alter the balance of microbiota in favour of protective bacteria and thus help to resolve uveitis. Such studies could form the basis for therapeutic modulation of diseases. In a recent study, in mice, it was demonstrated that signals from the gut microbiota activate retina specific T cells that are involved in autoimmune uveitis [23]. Such information would open up new strategies for therapy were the emphasis would be on restoring the diversity in the gut by antibiotic or specific drug use, by specific microbe introduction, by probiotic use and by fecal transplant therapy.

Ocular surface microbiome may also be responsible for eye diseases in man. Dong et al. [24] studied the microbiome of the healthy cornea and conjunctiva of four individuals and identified a core microbiome of 12 genera which included commensal, environmental, and opportunistic pathogenic bacteria. Whether the ocular microbiome exhibits dysbiosis with disease? Whether ocular microbiome is influenced by the gut microbiome? What mediates the cross-talk between the gut and ocular microbiomes? These are questions that need to be addressed to understand idiopathic infections of the eye. Research directed on the above lines would help to establish whether the gut microbiome influences ocular surface disease directly or indirectly by altering the ocular microbiome and probably also help to identify the causative organism.

## Conclusions

What seems to be amiss in these studies is an over emphasis on establishing core microbiomes rather than trying to ascertain the reasons for dysbiosis and methods to overcome dysbiosis. Further attempts to get at the relevance of dysbiosis by manipulating the microbiomes or by animals studies is also limiting. Above all, work on bacterial communities far outpaces that of viral and eukaryotic communities, although these agents are resident in the gut and could be the cause of several diseases. Finally evaluating diseases remote from the gut such as the eye, brain, kidney etc. would unfold the mysteries of the new organ the “microbiome”. It is only future studies that would either prove or disprove what Cho and Blaser [4] have said “—this (microbiome) is a frontier for human preventive medicine and for medical management of chronic diseases”.

## Abbreviations

CDI: *Clostridium difficile* infection
PSA: polysaccharide antigen A
